# Pre-assembled complexes and allosteric effects: parallels between eukaryotic phosphorylation cascades and membrane fusion during herpesviral entry

**DOI:** 10.1128/jvi.01704-24

**Published:** 2026-01-05

**Authors:** Gonzalo L. Gonzalez-Del Pino, Ekaterina E. Heldwein

**Affiliations:** 1 Department of Molecular Biology and Microbiology and Tufts NIH-IRACDA Program, Tufts University School of Medicine 12261, Boston, Massachusetts, USA; Indiana University Bloomington, Bloomington, Indiana, USA

**Keywords:** herpesvirus, *Herpesviridae*, HSV-1, glycoprotein, gB, gH/gL, gD, receptor, nectin-1, viral entry, membrane fusion, prefusion, postfusion, pre-activation, post-activation, MAPK, signaling cascade, signal transduction, allosteric

## Abstract

Unlike most enveloped viruses, *Herpesviridae* distribute cell entry functions across several viral envelope proteins. The prevailing model posits that, upon interaction with the target cell, the activating signal is transmitted from the receptor-binding to the fusion-mediating component in a signaling cascade that involves sequential interactions. However, herpesvirus entry proteins may form complexes throughout fusion. Here, we propose that—by analogy with certain eukaryotic signaling cascades—transmission of the activating signal involves pre-assembled complexes and allosteric effects.

## HERPESVIRUSES REQUIRE MULTIPLE GLYCOPROTEINS TO ENTER TARGET CELLS

Entry into a target cell is essential for a successful viral infection. Hence, viral entry machinery is exquisitely tuned to be deployed at the right place and time. For instance, it must be sensitive enough to be triggered by the appropriate host cell receptors, to avoid entry into the “wrong” host. It must also be robust enough not to be set off prematurely, locking the virus out of a potential host. Finally, it must be immunologically “quiet” enough to avoid triggering host defenses. All enveloped viruses enter host cells by fusing their lipid envelopes with a host cell membrane upon recognition of the cognate host receptor (1, 2). In most enveloped viruses, key entry functions—receptor binding and membrane fusion—are performed by a single glycosylated envelope protein (glycoprotein). A few notable examples are the human immunodeficiency virus envelope, influenza hemagglutinin, and severe acute respiratory syndrome coronavirus-2 Spike.

**Fig 1:**
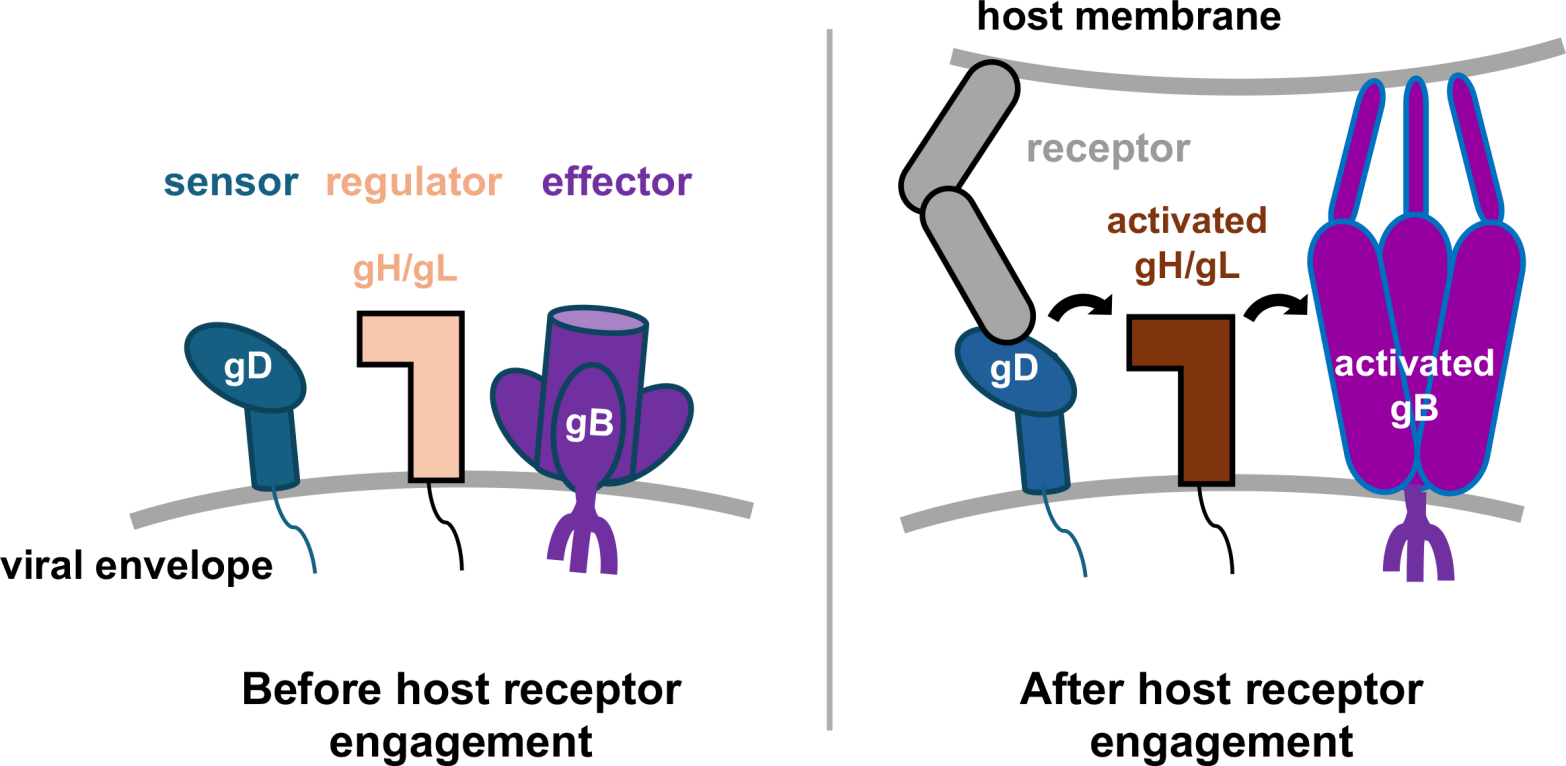
Linear cascade model of membrane fusion activation during herpesviral entry. Herpesviral cell entry requires three components: a sensor, a regulator, and an effector. In HSV, these are gD, gH/gL, and gB, respectively. Current models posit that these glycoproteins do not interact in the absence of a host receptor. Upon binding a cognate receptor, gD binds and activates gH/gL, which then binds and activates gB. gB refolds from its metastable prefusion form into an extended intermediate, initiating the membrane fusion process.

Some viral families, however, distribute these entry functions across two or more components. For example, *Orthoherpesviridae* (commonly known as herpesviruses)—a family of double-stranded-DNA, enveloped viruses that infect hosts from humans to reptiles for life—encode three different components that perform complementary roles: receptor-binding, regulatory, and fusogenic (3–6) (Fig. 1). The receptor-binding component varies across *Orthoherpesviridae* subfamilies, with some viruses even using different receptor-binding components for entry into different cell types (7), which is unsurprising given its “point-man” role. Upon binding its cognate receptor, the receptor-binding component transmits the activating signal to the regulatory component. In all *Orthoherpesviridae*, this role is fulfilled by the gH/gL complex that, in response to being activated, serves as the “middle-man” (8) to activate the fusogen gB – the “action-man”. gH/gL is highly conserved and has two structurally and functionally distinct regions that fulfill two distinct roles: binding a viral receptor-binding component (or the host receptor) and triggering gB. gB is the membrane fusogen undergoing large conformational rearrangements to bring apposed membranes together. gB is highly conserved across *Orthoherpesviridae* in sequence and structure (9). But how these glycoproteins come together to bring about membrane fusion is a key unanswered question in the field.

The three components required for host entry across *Orthoherpesviridae*—receptor-binding, regulatory, and fusogenic—must interact to accomplish their joint function. In the prototypical herpes simplex viruses (HSV)—the main focus of this review—these components are gD, gH/gL, and gB, respectively. The prevailing model for fusion activation posits that these components sequentially bind and activate each other (Fig. 1). Specifically, upon receptor binding, gD (the sensor glycoprotein) undergoes a conformational change that allows it to bind and activate gH/gL. Activated gH/gL (the regulator complex) then, in turn, binds and activates gB (the effector glycoprotein). But how the activating signal is transmitted from receptor-binding to fusogenic components is yet unclear.

## THE HERPESVIRUS ENTRY GLYCOPROTEINS ARE CONFORMATIONALLY DYNAMIC AND LABILE

While the exact process of sequential activation of the herpesviral entry glycoproteins remains a mystery, it undoubtedly involves conformational rearrangements brought about by receptor binding. Best studied are the conformational changes in gD, in which binding the host receptor causes the displacement and release of its C-terminal profusion domain (PFD), which somehow activates gH/gL (10, 11). Crystal structures of gD in unliganded (12) and ligand-bound (13–15) states have been determined. In the unliganded state, the PFD shields the receptor-binding site (12). Its displacement by receptor-binding suggests that the PFD may sample multiple conformations in its basal state, some of which would allow the receptor to initially engage with gD. It remains unclear, however, what the basal state of gD is on the virion surface.

Little is known about conformational rearrangements in gH/gL. Until recently, all available structures of gH/gL homologs represented one conformation (16–23). A recent study from our laboratory uncovered significant conformational dynamics in the gH/gL ectodomain (24). Our cryo-EM analysis showed that gH/gL exists in a conformational continuum, ranging from a state that may represent the basal, or pre-activation state of gH/gL to another state that may represent its triggered, or activated, state. The transition from the pre-activation to the activated state of gH/gL coincides with conformational changes at the N- and C-terminal regions of gH. Given that these conformational changes occur near the putative gD- and gB-binding sites in gH/gL, we hypothesized that interaction with a receptor-bound gD causes the release of the gH N terminus. This event, in turn, causes allosteric conformational changes in a C-terminal switch element of gH that, given its location, may be involved in the activation of gB. This suggests that gH/gL is kept in a sensitive, metastable state prior to host-receptor binding.

Like all viral fusogens, gB initially folds into a relatively high-energy, prefusion form and, after being activated by gH/gL, refolds into a low-energy, postfusion form. These thermodynamically favorable conformational changes are thought to provide the free energy required for merging the viral envelope with an apposed host membrane (1, 2). The structures of gB homologs in pre- (25–28) and postfusion (29–34) forms have yielded snapshots of the initial and final conformations, and the large conformational changes that accompany refolding. Structures of likely refolding intermediates have also been reported (27).

It is worth pointing out the time and effort that was required to obtain the structures of the basal, pre-activation/prefusion states of the herpesviral entry glycoproteins due to their labile nature. For example, obtaining the structure of unliganded gD with ordered PFD required stabilization of the gD dimer by an intermolecular disulfide (12). Similarly, obtaining the structure of gH/gL in its pre-activation state (18, 24) required the use of crosslinking agents and monoclonal antibodies 15 years after the first structure was determined (18, 24). Finally, the first structure of the gB ectodomain revealed the fusogen in its triggered, postfusion form (29). It took nearly 15 years of work and cutting-edge cryoelectron microscopy and tomography to obtain the first low-resolution structure of its basal, prefusion form (25).

These observations raise a tantalizing question: if the components in the herpesvirus entry cascade are conformationally dynamic and labile, how are they stabilized in their basal, fusion-competent states on the viral envelope or cell surface? The presence of a lipid membrane likely plays an important role because disruption of membrane interactions, e.g., removal of a membrane anchor or treatment with detergent, triggers conversion of gB into the postfusion form (29–32, 34). Additionally, the membrane-interacting cytoplasmic tail of gB has an inhibitory effect on fusion (9, 32). However, membrane interactions do not fully account for the stabilization of gB since gB is found in both its prefusion and postfusion conformations on virions (35, 36) and cells overexpressing gB (25). One plausible hypothesis is that stabilization of entry glycoproteins is achieved through the formation of complexes.

## HERPESVIRAL ENTRY GLYCOPROTEINS MAY FORM COMPLEXES BEFORE AND DURING FUSION

Herpesvirus glycoproteins walk the tightrope between sensitivity and robustness to trigger fusion in the appropriate contexts, chiefly through manipulation of binding site accessibility and complex stability throughout the entry process. For example, binding of gD to one of its receptors, nectin-1, is regulated by its C-terminal PFD (15). In the unliganded gD, the PFD is bound to the immunoglobulin-like globular domain of gD and occludes the nectin-1-binding site (12), which results in a micromolar binding affinity (12, 37). Removal of the PFD increases the binding affinity ~100-fold.

gD has been proposed to dimerize on the surface of the virion (38, 39). Indeed, the structure of unliganded dimeric gD has been determined (12). The PFD, which occludes the glycoprotein’s receptor-binding surfaces, contributes to the dimerization interface (12). While it is yet unclear whether gD dimerization is functionally relevant for the fusion process, if it were, it could regulate an early step of entry. The gD ectodomain does not bind the gH/gL ectodomain in solution (40). However, gD-gH/gL binding becomes detectable when gD is tethered to the surface of a microfluidic chip using an antibody (40). Since antibodies are bivalent, this observation could imply that gD dimerization promotes binding to gH/gL, possibly by increasing adjacent binding sites through avidity. If gD dimerization inhibits its binding to receptor by obstructing the receptor-binding site, while at the same time promoting binding to gH/gL, this could mean that in the absence of a host receptor, gD and gH/gL interact on a virion or cell surface.

This hypothesis is bolstered by the fact that in other subfamilies of *Orthoherpesviridae*, gH/gL forms stable complexes with the receptor-binding analogs of gD. For example, in human cytomegalovirus (HCMV), gH/gL is covalently bound (through a disulfide) to either gO or UL128/130/131 (22, 41) whereas in Epstein-Barr virus, gH/gL forms a noncovalent complex with gp42 (42). Furthermore, the HCMV gH/gL/UL128/UL130/UL131 can form dimers in solution when bound to a cognate receptor (21, 43), which suggests that dimerization of a receptor-binding protein complex could be important across herpesviruses.

Importantly, under biologically relevant conditions, herpesvirus entry glycoprotein interactions occur in the context of full-length proteins containing intact membrane-spanning and intraviral (cytoplasmic) regions, in addition to the ectodomains. gD, gH, and gB are single-pass transmembrane proteins, and their ectodomains (ECTO), transmembrane (TM) anchors, and cytoplasmic tails (CT) all contribute to the fusion process. While the ECTOs are involved in binding to the host cell and facilitating the membrane merger, the TMs and CTs contain important regulatory regions. For example, the gH CT activates the fusion activity of gB, whereas the gB CT is inhibitory (44, 45). Using a split nanoluciferase assay, our laboratory previously demonstrated that the TMs and CTs of gD, gH, and gB contribute to their respective interactions (46, 47). These experiments also revealed that the three components may interact (or are, at least, in proximity to each other) before and during membrane fusion. This is consistent with immunoprecipitation experiments in HCMV, where gB was pulled down when precipitating gH/gL complexes from virions in the absence of any host receptors (48). Finally, cryoelectron tomography (cryoET) of HCMV particles found a small yet significant proportion of gB to be associated with gH/gL on the viral surface (35). Notably, none of the gB associated with gH/gL was found in its postfusion form by cryoET (35).

A lack of detectable interactions between the soluble ectodomains of HSV gD, gB, and gH/gL raises two possibilities. First, the transmembrane and cytoplasmic regions may contribute significantly to glycoprotein interactions. Indeed, the results of the split nanoluciferase experiments point to the importance of the transmembrane and cytoplasmic regions to glycoprotein proximity (if not interactions) and membrane fusion (47). This highlights the role of avidity—the cumulative effect of multiple interactions across a protein-protein interface—in keeping weakly bound protein domains together across a larger complex. Second, the purified, soluble ectodomains may adopt conformations that have lower affinity for their partners. In support of this idea, the wild-type, soluble gB ectodomain adopts the postfusion conformation after purification (29). Likewise, the soluble gH/gL ectodomain appears to sample many different conformational states across a pre-activation-to-post-activation continuum (18, 24). If this is so, we would expect their prefusion or pre-activation conformations to be more likely to interact than their postfusion/post-activation counterparts. This also suggests that the ectodomains may dominate interactions among the entry glycoproteins before receptor engagement.

The labile nature of the prefusion and pre-activation states of gB and gH/gL takes us back to the question posed earlier in this review: how are these glycoproteins kept primed for activation without being triggered prematurely? Large proportions of free gB are found in the postfusion conformation on the surface of virions across the human *Orthoherpesviridae* subfamilies (35, 36). These triggered gB molecules have been proposed to serve as decoys meant to soak up neutralizing antibodies, leaving the glycoproteins still in the lower-profile prefusion conformation able to fuse the viral envelope with a host membrane. Tomography studies of viral surfaces have not yet achieved resolutions capable of distinguishing between pre-activation and post-activation gH/gL particles.

Although the formation of multicomponent complexes could explain how the labile forms of entry glycoproteins are stabilized, it leaves open the question of how the signal from the receptor is transmitted to gB to promote membrane fusion. While the answer is not yet clear, clues can be gleaned from other conceptually similar systems where this question has been better addressed. One such system is the mitogen-activated protein kinase (MAPK) cascade.

## THE MAPK CASCADE IS MORE THAN A LINEAR SIGNALING CASCADE

The ability to sense changes in an environment and respond accordingly is a fundamental property of many biological systems. The underlying mechanism is termed signal transduction because, within a multicomponent system, an environmental signal is first detected by one component, which transmits (or transduces) the signal to its downstream partner, and so on, until, at the end of this cascade of signaling events, an output is produced. Some of the best-characterized examples of such signaling pathways are phosphorylation cascades, in which—in response to an extracellular hormone or intracellular metabolite—a series of kinases transfer a phosphate from ATP to their immediate downstream targets, sequentially activating the enzymatic activity of the latter so that they can, in turn, phosphorylate their respective substrates.

**Fig 2:**
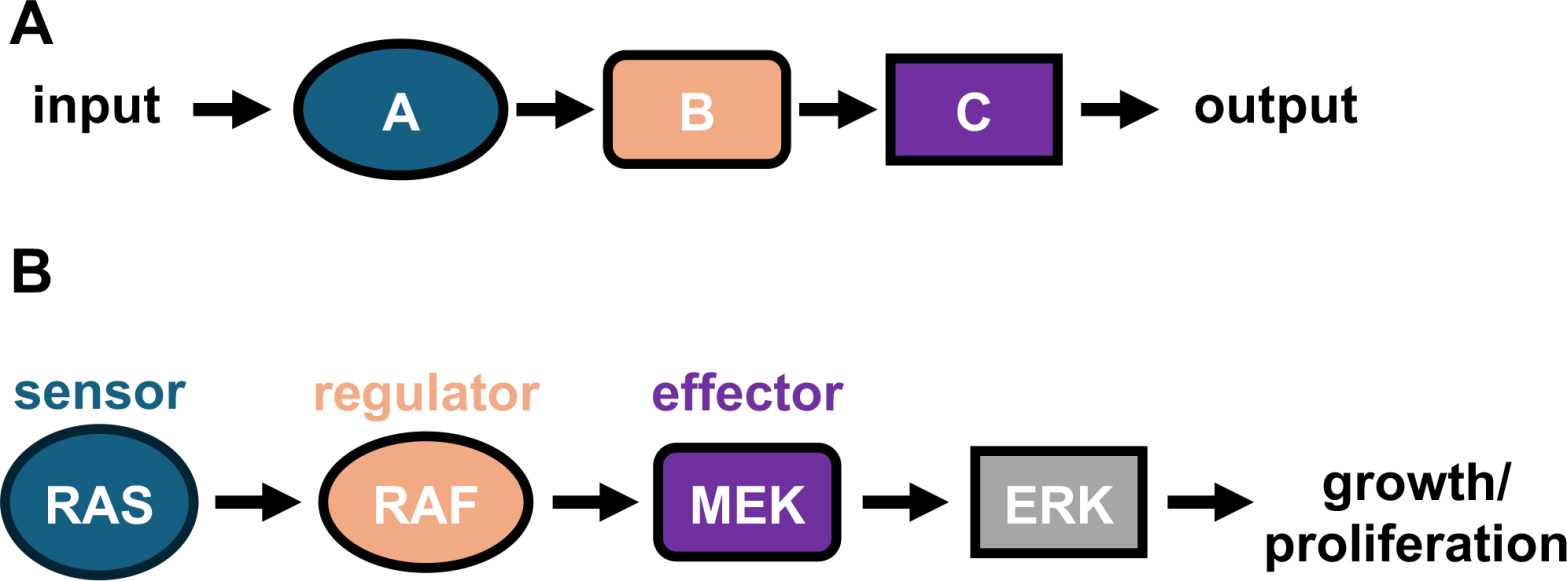
Signal transduction through the MAPK cascade. (**A**) A linear cascade view of a generic signal transduction pathway. (**B**) A traditional linear view of MAPK signal transduction. The small GTPase RAS detects cues from extracellular receptors and activates the MAP kinase RAF, which phosphorylates and activates its downstream target, MEK. MEK then phosphorylates and activates ERK, leading to growth and/or proliferation.

Our initial understanding of phosphorylation cascades resulted from genetic and phenotypic studies, which posited a linear order of events in many transduction pathways (Fig. 2A). This view of signaling pathways has two main shortcomings, however. First, it oversimplifies the complex milieu of a cell where multiple pathways may intersect, pleiotropic genes are present, and cascades may have built-in redundancy. Furthermore, it tends to overlook the protein nature of the cascade components that must find their downstream partners and perform their function (phosphorylation, cleavage, dimerization, etc.) to transmit the signal in the crowded environment of the cell. The subsequent use of biochemical and structural approaches has helped fill in some of these gaps, expanding a simplified, linear representation of the signaling cascades to produce a more accurate mechanistic model.

The MAPK cascade is one of the best-studied signaling cascades in biology and a major pathway used by eukaryotes to respond to growth, proliferation, and differentiation signals (49). Because of its control of these cellular programs, mutations in MAPK enzymes cause many cancers and other diseases relevant to human health. The simplified, linear diagram (Fig. 2B) belies the remarkable complexity of the regulation and pharmacology of this pathway. For example, there are three RAF family kinases with similar but non-overlapping functions in humans (ARAF, BRAF, CRAF) (50). Moreover, other kinases and pseudokinases bind to and modulate the activities of RAF, MEK, and ERK (51). These confounding factors have made it difficult to effectively target this pathway with therapeutics.

Over the past decade, a clearer picture of the step-by-step mechanism of the MAPK cascade has emerged largely due to the use of sensitive biophysical, biochemical, and structural approaches. The prevailing dogma was that, in the absence of an extracellular signal, RAF and MEK components existed as inactive monomers in the cytosol. This dogma was challenged by pulldown experiments of differentiated and proliferating cells that revealed that one RAF family kinase, BRAF, is, instead, constitutively bound to its downstream target, MEK1, as well as a dimeric 14-3-3 chaperone, even in the absence of an activating cue (52, 53). This finding was bolstered by subsequent structures of BRAF/MEK1 complexes not only in an active dimer-of-heterodimers (RAF_2_/MEK_2_) configuration but also in a primed, autoinhibited BRAF/MEK1/14-3-3 complex (54).

Importantly, a better understanding of the assemblies and rearrangements involved in signal transduction has translated to better rational drug design targeting the MAPK cascade. A follow-up study showed that the actual target for the majority of MEK1 inhibitors used in the clinic or in clinical trials is the BRAF/MEK1 dimer and not MEK1 alone (55). Furthermore, the potency of RAF and MEK inhibitors varies depending on which member of the RAF family (ARAF/BRAF/CRAF) is bound to MEK (56). MEK1 inhibitors thus target MEK not directly but rather allosterically by inhibiting RAF activation of MEK in the RAF/MEK complex. Specifically, some MEK1 inhibitors prevent the activation of MEK1 by inhibiting full phosphorylation of MEK1 activation loop residues by RAF or by preventing the release of phosphorylated MEK1 from RAF, both of which block its downstream function on ERK.

**Fig 3:**
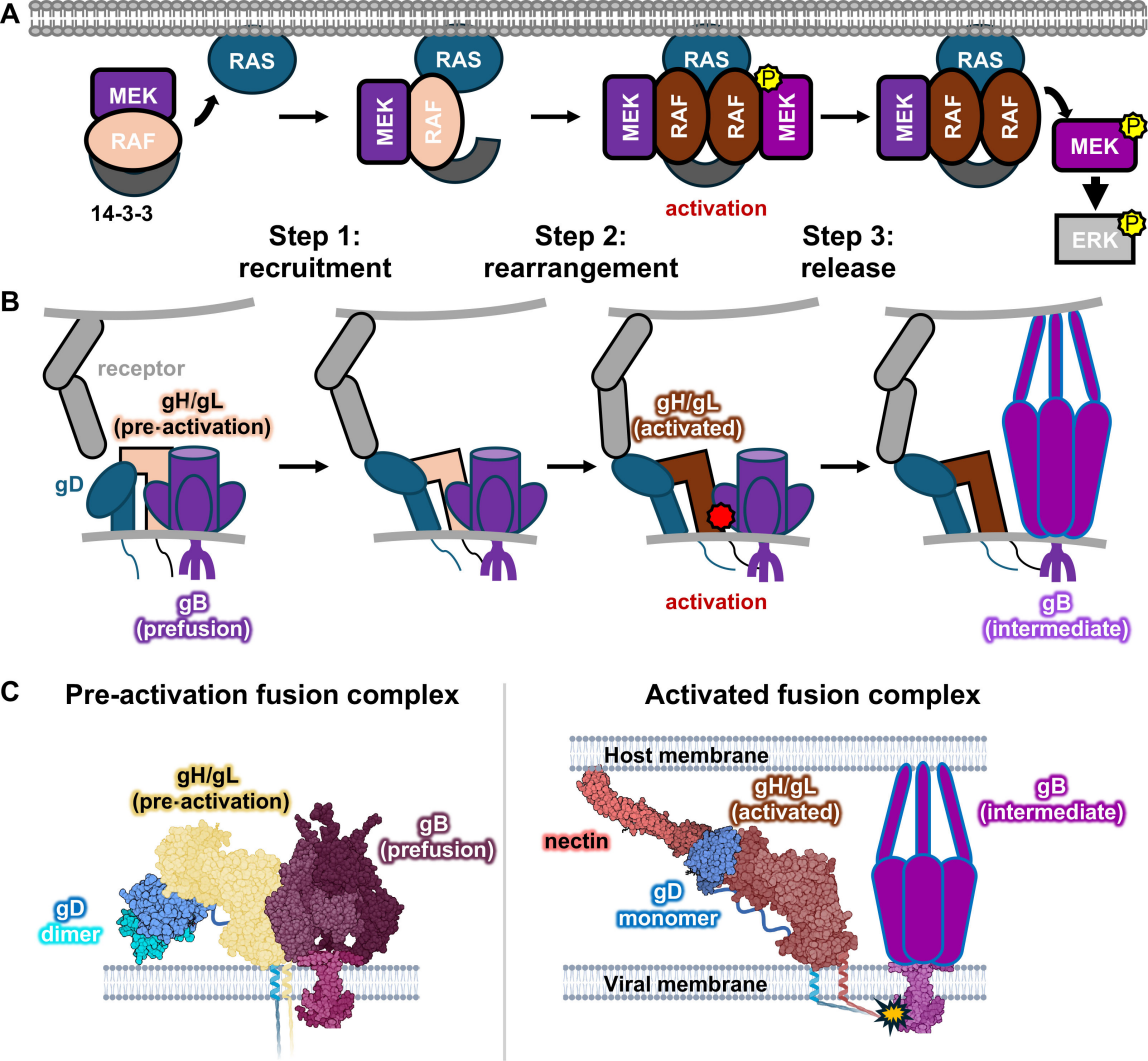
Pre-assembled complex models of the kinase cascade activation in MAPK signaling and membrane fusion activation in HSV entry. (**A**) A revised model of MAPK signaling. RAF and MEK form an autoinhibited complex prior to recruitment by RAS. Once recruited by RAS, the RAF/MEK complex undergoes a rearrangement, forming a phosphorylation “factory” at the plasma membrane. Phosphorylated MEK dissociates from RAF and phosphorylates ERK. (**B**) Once the virus comes in contact with a host cell, gD is activated. The activated gD globular domain adopts a conformation capable of perturbing gH/gL, presumably through the displacement of the PFD by host receptor. gH/gL is then activated by the gD-nectin complex, rearranging into its active conformation. This conformational change allosterically transduces the signal from the adaptor-binding N terminus to the fusogen-binding C terminus. gH/gL and gB then dissociate, freeing gB to undergo fusogenic refolding. (**C**) Structural models for the hypothetical HSV fusion complex in its primed and activated configurations. PDB IDs: gD dimer: 1L2G, prefusion gB: 6Z9M, nectin-gD complex: 4MYW, activated gH/gL: 3M1C.

Although having a pre-assembled BRAF/MEK1 signaling complex may seem counterintuitive, for a time-sensitive process, such as cellular response to growth/differentiation signals, having a primed pre-assembled machine would allow for a faster downstream effect and is, therefore, beneficial if not crucial. But, if the RAF/MEK signaling complex is not assembled in response to a signal and is, instead, pre-assembled, how is the signal transmitted down the cascade? In the MAPK case, this process involves three steps. First, the pre-assembled RAF/MEK complex is recruited from the cytoplasm to bind the membrane-associated small GTPase RAS (Fig. 3A, step 1). Next, the RAF/MEK complex rearranges to form the enzymatically active dimer-of-heterodimers RAF_2_/MEK_2_ complex (Fig. 3A, step 2). Finally, phosphorylated, enzymatically active MEK dissociates from RAF and proceeds to phosphorylate ERK, with concomitant changes in cellular response (Fig. 3A, step 3).

The parallels between these two systems may not be immediately apparent. The MAPK cascade is a sequential protein phosphorylation process, whereas herpesvirus host entry is a mechanical process that uses proteins and physical forces to merge lipid bilayers. Nonetheless, knowledge from MAPK signaling cascades can provide useful analogies for improving our understanding of other multicomponent systems that involve sequential protein interactions, i.e., membrane fusion by herpesviruses. Below, we detail how the model of signal transmission within a pre-assembled multicomponent complex may apply to entry of herpesviruses, using the prototypical HSV as an example.

## A PRE-ASSEMBLED COMPLEX MODEL OF MEMBRANE FUSION ACTIVATION IN HERPESVIRAL ENTRY

The sequential process of herpesvirus entry may resemble the three-step process of a well-regulated signal transduction pathway, such as the MAPK cascade introduced above: recruitment, rearrangement, and release (Fig. 3). To begin with, just as RAF and MEK form an inactive complex in the cytoplasm (Fig. 3A, step 1), gD, gH/gL, and gB interact on the surface of a virion (Fig. 3B, step 1). Then, just as in the MAPK cascade where the RAF/MEK dimer must be recruited to RAS on the cytoplasmic side of the plasma membrane (Fig. 3A, step 1), to initiate herpesvirus entry, gD binds one of its receptors on the surface of the host cell, which sets in motion the signaling cascade within the herpesvirus entry machinery (Fig. 3B, step 1). Next, similarly to a rearrangement in the RAF/MEK complex that activates it (Fig. 3A, step 2), once a host receptor is engaged, conformational changes in gD cause conformational changes in gH/gL that allosterically transmit the fusion-activating signal from gD to gB (Fig. 3B, step 2). This signal may lead to the release of gB by activated gH/gL, freeing the fusogen to refold from its labile prefusion conformation through the extended intermediate to the stable postfusion conformation, ultimately achieving the merger of viral and host membranes (Fig. 3B, step 3). This release has parallels with the dissociation of the RAF/MEK complex, which frees phosphorylated MEK to activate ERK (Fig. 3A, step 3). Although the structure of the complex has not yet been determined, we have put together a hypothetical model of the gD-gH/gL-gB complex that may exist on the surface of a virion or an infected cell from structures of the glycoproteins involved in different conformation and binding states (Fig. 3C).

Having all components of the entry machinery together in a primed, autoinhibited complex also satisfies the viral entry process requirements of sensitivity, robustness, and immunoevasion. By having the host-sensing, signal-transducing, and membrane-fusing components in one place, the initial step of finding binding partners is bypassed, allowing for a near-instantaneous response once the host-receptor binding protein has engaged its target. At the same time, a preformed complex adds a layer of stability to these dynamic proteins by having the prefusion/pre-activation forms buttress each other. Finally, having the entry complex assembled in the endoplasmic reticulum of a host cell and transported together to the cell surface for virion budding means that the absolute number of glycoproteins on the viral surface can be lower. Fewer glycoproteins on the viral surface means that there are fewer targets for antibody recognition and neutralization. Recent cryoelectron tomography experiments have indicated that the glycoproteins on the surface of *Herpesviridae* tend to cluster together, further increasing the likelihood that multiple fusion complexes will be close enough to efficiently fuse lipid bilayers (36).

There are still many unanswered questions in herpesvirus entry, some of which might be addressed by structural and biochemical approaches. The structures of the glycoprotein complexes required for activation of the fusion program (gD-gH/gL-gB in HSV, gH/gL-gB in beta- and gammaherpesviruses) are vital targets for both basic and translational virology. If, as we suspect, the pre-activation/prefusion conformations of the herpesviral glycoproteins interact and keep each other in a primed, autoinhibited complex, stabilizing these conformations in each component of the fusion machinery should facilitate structure determination efforts. This has precedent in the structural characterization of the MAPK pathway. Since the discovery that phosphorylation of two serines in the MEK1 activation loop by BRAF decreases the affinity between these two kinases by more than 100-fold, mutation of these serines to alanines has allowed purification of the inactive BRAF/MEK1/14-3-3 complex (54). Indeed, several groups have used mutagenesis and, in one case, small molecules, to stabilize the prefusion conformation of gB (25–28). The next goal should be stabilizing gH/gL and gB in their pre-activation/prefusion conformations using mutations, small molecules, or antibody-binding to induce complex-permissive conformational states to capture and characterize the steps in the fusion activation cascade.

Finally, if the pre-assembled complex model for herpesvirus entry is correct, the primed, autoinhibited complex on the viral surface should be the main objective for the development of prophylactics and therapeutics. Raising antibodies against the full complex rather than individual proteins may lead to more effective protective antibodies, similarly to how the BRAF/MEK1 dimer appears to be the main target of allosteric MEK1 inhibitors rather than free MEK1 in solution.
